# Ubiquitin-specific peptidase 22 functions and its involvement in disease

**DOI:** 10.18632/oncotarget.8602

**Published:** 2016-04-05

**Authors:** Johanna Melo-Cardenas, Yusi Zhang, Donna D. Zhang, Deyu Fang

**Affiliations:** ^1^ Department of Pathology, Northwestern University Feinberg School of Medicine, Chicago, IL, USA; ^2^ Department of Pharmacology and Toxicology, University of Arizona, Tucson, AZ, USA

**Keywords:** USP22, deubiquitylation, cancer, oncogene, ataxia

## Abstract

Deubiquitylases remove ubiquitin moieties from different substrates to regulate protein activity and cell homeostasis. Since this posttranslational modification plays a role in several different cellular functions, its deregulation has been associated with different pathologies. Aberrant expression of the Ubiquitin-Specific Peptidase 22 (USP22) has been associated with poor cancer prognosis and neurological disorders. However, little is known about USP22 role in these pathologies or in normal physiology. This review summarizes the current knowledge about USP22 function from yeast to human and provides an overview of the possible mechanisms by which USP22 is emerging as a potential oncogene.

## INTRODUCTION

Cells have different mechanisms to control the activity of proteins such as posttranslational modifications. These modifications lead to protein activation, inactivation, compartmental targeting, creation of recognition sites and degradation. As a result of these posttranslational modifications, the cells quickly respond to extracellular clues, stress or DNA damage to maintain intracellular homeostasis. One of these post-translational modifications is ubiquitylation, which is the conjugation of ubiquitin, a small protein of 8.5 kDa [1, 2], at specific lysine (K) or amino-terminal methionine (M1) residues on the target proteins. As indicated by its name, this protein is expressed ubiquitously in all eukaryotic cells. Three different enzymes carry out the ubiquitylation process: ubiquitin-activating enzymes (also known as E1s), ubiquitin-conjugating enzymes (E2s) and ubiquitin ligases (E3) [3]. Ubiquitin can be added to the target proteins as a single molecule (monoubiquitylation) or as chains (polyubiquitylation).

Different lysine residues on ubiquitin are used for conjugation. K48 and K63 are the best understood. K48-ubiquitin chains are mostly associated with targeting of proteins for destruction by the proteasome but they also have a role in transcription. K63-ubiquitin chains are associated with the recruitment of other proteins that are involved in endocytic trafficking, DNA repair and signal transduction [4, 5]. Linear ubiquitylation (M1-linked ubiquitin) has been shown to be important for the nuclear factor-κB and cell survival [6]. Overall, protein ubiquitylation is important for: biogenesis, cell cycle, apoptosis, immunity, transcription, differentiation, development and response to stress.

The deubiquitylases (DUBs) oppose the activity of the ubiquitylating enzymes. DUBs are cysteine proteases that remove ubiquitin from the target protein by cleaving the amide bond between the two proteins. They are highly specific and affect all the roles described for ubiquitylating enzymes and produce monoubiquitin molecules for recycling [7]. In humans, about 100 DUBs have been identified and these are grouped into 6 families: ovarian-tumor proteases (OTUs), monocyte chemotactic protein-induced protein (MCPIP), Machado-Joseph disease protein domain proteases, JAMM/MPN domain-associated metallopeptidases (JAMMs), ubiquitin carboxy-terminal hydrolases (UCHs) and ubiquitin-specific proteases (USPs) [7]. USPs are the largest DUB family containing more than half of the DUBs identified. Members of this family are highly conserved and contain three subdomains resembling the fingers of a right hand, the thumb and the palm.

Due to the multiple roles DUBs have in cell homeostasis, their deregulation has been found in cancer and neurological disorders [8, 9]. DUBs have been shown to regulate p53 activity, WNT, EGFR, NF-κB pathways, cell cycle progression, apoptosis and response to DNA damage. This review will focus on USP22, which has recently emerged as an important DUB that leads to cancer progression in several types of cancer. USP22 deubiquitylates histone and non-histone substrates and has been associated with cancer progression and spinocerebellar ataxia.

### What is USP22?

The Ubiquitin-Specific Peptidase 22 (USP22) belongs to the largest subfamily of deubiquitylases, the ubiquitin-specific processing proteases (USPs), which in yeast are called ubiquitin-binding proteins (UBPs). USP22 is highly conserved from yeast to vertebrates. USP22 in yeast, called Ubp8, has been shown to bind to Sgf73, Sgf11 and Sus1 to form the deubiquitylase module (DUBm) of the Spt-Ada-Gcn5 Acetyl transferase complex (SAGA). The SAGA complex enhances transcriptional activation and facilitates elongation by RNA polymerase II through its histone acetylation and deubiquitylation activities [10].

## USP22 STRUCTURE AND SAGA DEUBIQUITYLASE MODULE FORMATION

USP22/Ubp8 is composed of two different domains, the zinc finger (ZnF) at the N-terminal and the catalytic domain at the C-terminal [11]. Analysis of crystal structures of Ubp8 shows that the ZnF domain lacks the ubiquitin-binding pocket observed in other USPs. Therefore, instead of binding ubiquitin, this domain is important for the binding to Sus1, Sgf11 and Sgf73, which holds the DUBm together in yeast [12, 13].

The catalytic domain of Ubp8 it is composed of three subdomains: Fingers, Palm and Thumb. The cysteine protease active site of the catalytic domain is found between the Palm and the Thumb, which unlike other USPs, is catalytically competent even in the absence of ubiquitin. Sgf11 and Sgf73 activate Ubp8 catalytic activity and bind nucleosomes bringing the DUBm into close proximity with the histones [14, 15].

Human orthologs of Sgf11, Sus1 and Sgf73, named ATXN7L3, ENY2 and ATXN7 respectively, have been found in the DUBm of human SAGA. ATXN7L3 (composed of 354 amino acids) has an additional SCA7 domain, which is absent in yeast Sgf11 containing only 99 amino acids [15]. ATXN7L3, ENY2 and ATXN7 are critical for USP22 activity [16]. Paralog proteins of ATXN7 (ATXN7L1 and ATXN7L2) were also found as part of the human SAGA components when USP22 was immunoprecipitated [16, 17]. However, their function in the DUBm has not been explored.

Different stable USP22-containing complexes have been shown to form *in vitro* and *in vivo* some of them lacking ATXN7 or ENY2 [16, 18]. Although the role of these DUBm SAGA-independent complexes are not yet fully studied, they seem to be important for mRNA related processes. Lim et al [19] showed that DUBm separation from SAGA complex was necessary for mRNA export in yeast but no studies have addressed whether this also occurs in mammalian cells.

### USP22 substrates

#### Histones

USP22/Ubp8 substrates now identified are histones and non-histone proteins (Table 1). It has been shown that Ubp8 deubiquitylates H2Bub on lysine 123 (H2BK123) and USP22 on H2BK120. This lead to increased transcription of several genes. Mutations on H2BK123 that remove the ubiquitylation site or impair Ubp8 activity, lead to lower transcription levels [20]. This ubiquitylated or deubiquitylated state of H2B changes the methylation of histone 3 (H3) at specific sites, as well as the number of methylations, which is also important during transcription [21].

Ubp8 activity on H2B was mostly observed at the transcriptional start sites where a trimethylation mark was present in H3K4 (H3K4m3). Removal of the ubiquitin moiety on H2BK123 by Ubp8 has been shown to allow the recruitment of C-terminal kinase 1 (CtK1) resulting in the phosphorylation of the RNA-polymerase II and initiation of transcription [22].

Histone H2B deubiquitylation by USP22/Ubp8 has been shown to occur at the transcribed regions of target genes [16, 23]. Studies performed in Drosophila showed that nonstop and dSgf11 are required for the expression of silenced genes associated with heterochromatin [24]. Deubiquitylation of H2B by Ubp8 has also been associated with DNA damage repair after UV irradiation [25].

Histone H2Aub was also identified as a USP22 substrate *in vitro* [26]. H2A ubiquitylation by the Polycomb repressive group has been associated with repression of genes involved in differentiation [27]. However, the functional impact of USP22 deubiquitylation of H2A has not yet been studied *in vivo*.

**Table 1:** USP22 substrates and functions

Substrate	Species	Functions	References
**Histone H2B**	Yeast and human	Transcription activation Recruitment of CtK1 that phosphorylates RNA-pol II	45, 20, 16, 22, 24, 25
**Histone H2A**	Yeast and human	Unknown	24, 26
**TRF1**	Human	Protein stabilization regulating telomere length	29
**SIRT1**	Human	Protein stabilization leading to inhibition of cell death	30, 31
**Cyclin B1**	Human	Protein stabilization promoting nuclear accumulation and cell cycle progression	32
**FBP1**	Human	Removal of Lys-63 allowing FBP1 binding to target loci	33
**Hes1**	Mouse	Protein stabilization regulating neuronal differentiation	34
**RCAN1**	Human	Protein stabilization	35
**NFATc2**	Human	Protein stabilization regulating IL-2 production	36
**COX-2**	Human	Protein stabilization	37
**Snf1**	Yeast	Protein stabilization allowing for transcription of glucose-repressed genes	38

#### Non-histone substrates

The notion that USP22 might also have non-histones substrates came from genetic studies in drosophila where mutations in nonstop lead to accumulation of ubiquitylated proteins in larval tissue [28]. Non-histone substrates identified so far include the telomeric repeat binding factor 1 (TRF1) [29], sirtuin 1 (SIRT1) [30, 31], cyclin B1 [32], the far upstream element -FUSE- binding protein 1 (FBP1) [33], hairy and enhancer of split 1 (Hes1) [34], regulator of calcineurin 1 (RCAN1) [35], nuclear factor of activated T-cells (NFAT) [36], cyclooxygenase 2 (COX-2) [37] and sucrose-nonfermenting 1 (Snf1) [38].

In most of the cases, USP22 interaction with these substrates leads to protein stabilization and inhibition of degradation by the proteasome. For example, USP22 binding to TRF1, a protein that regulates telomere length, induces TRF1 protein stability. Cells depleted of USP22 have low TRF1 levels and present chromosomal abnormalities that lead to cell death [29]. USP22 also increases SIRT1 protein stability, which leads to the suppression of p53 transcriptional activity and inhibition of cell death [30, 31]. USP22 binding to Cyclin B1, promotes cyclin B1 accumulation in the nucleus and inhibits its degradation [32]. Interestingly, USP22 effect on FBP1, a transcriptional regulator, does not change FBP1 protein stability. Instead, USP22 increases FBP1 recruitment to target loci by deubiquitylation of K63 [33].

Other proteins have been shown to bind USP22 that are not contained in the SAGA complex. These proteins are involved in transcription and mRNA processing as well as proteins that have not been well characterized [17]. USP22 has been shown to be important for the processing of IRF1, POLR2F and GBP2 mRNA transcripts after IFNγ-stimulation [39]. In prostate cancer, USP22 overexpression lead to increased androgen receptor splicing [40]. These data open the possibility that USP22 either alone or with its DUBm partners may play a role in mRNA processing.

### USP22 regulation

Regulation of USP22 activity is still poorly understood. At the transcriptional level, Sp1 and PKA/CREB have been reported to bind to *USP22* promoter and regulate *USP22* transcription [41, 42].

At the posttranscriptional level, c-MYC has been shown to increase USP22 protein but not mRNA levels. c-Myc, through USP22, also increased SIRT1 protein levels which in turn deacetylated c-Myc to enhance its transcriptional activity [31]. SIRT1 decreases the acetylation state of USP22 and the other SAGA components to decrease their activity [43]. Acetylation of USP22 on lysine 129 (K129) regulates USP22 deubiquitylase activity and its association with the SAGA complex. Absence of K129 acetylation, as demonstrated by mutating of K to arginine (R), leads to decreased USP22 activity. However, mutations mimicking an acetylated state (K129Q) did not increase USP22 activity compared to basal levels in-vitro.

Other posttranscriptional modifications of USP22 have been also identified. Phosphorylation of USP22 at T147 and S237 by cyclin-dependent kinase 1 (CDK1) was shown to activate USP22 to deubiquitylate cyclin B1. These phosphorylated forms were specifically found in G2/M phase but not in G1 phase. Lastly, ubiquitylation of USP22 mediated by the anaphase promoter complex/cyclosome (APC/C) induces USP22 protein degradation during the cell cycle [32].

## PHYSIOLOGICAL FUNCTIONS OF USP22

USP22 functions described so far include the regulation of genes and proteins involved in metabolism, cell cycle and development. In yeast, deletion of Ubp8 leads to an extended lifespan [44] and a slight decrease of growth in galactose and ethanol/glycerol media. This is accompanied by a decrease in the mRNA levels of GAL1, GAL10 and ADH2 genes [45]. Upb8 null strains also showed decreased resistance to γ-radiation, and desiccation [46, 47].

In drosophila, nonstop expression is observed in the cytoplasm of cells located in the central brain, optic lobe, lamina precursor and glial cells [28]. Nonstop deletion is lethal and disruption of one allele leads defects in development and migration of glial cells, which results in an impaired visual system [48]. Nonstop was also found to counteract gene silencing in a model of position effect variegation [24]. Studies of SAGA occupancy performed in drosophila showed different binding profiles depending on the tissue analyzed. In embryonic muscle, SAGA was found to localize in almost 2000 genes. But in embryonic neurons, it was found in less than 600 genes most of which overlapped with the ones found in embryonic muscle. SAGA was also found to associate with different transcription factors that most likely directed SAGA into specific sets of genes [49].

In mouse and human tissues, Usp22/USP22 is ubiquitously expressed. During early developmental stages in mice, Usp22 expression is detected at E4.5 and peaks at E11.5 [50]. Genetic deletion of *Usp22* leads to early mortality with embryos not surviving beyond E10.5. These embryos presented retardation, small size and increased apoptosis compared to wild type or heterozygous littermates [30]. Mice with reduced Usp22 levels are viable but show growth retardation with impaired differentiation of the brain and the small intestine [51].

Little is known about the role of Usp22 in normal tissues. Few studies have explored the role of Usp22 during development. One study showed that in murine stem cells *Usp22* mRNA and protein levels increase when embryonic stem cells (ESCs) differentiated into embryoid bodies (EBs). Usp22 expression was associated with decreased expression of Sox2, a transcription factor essential to maintain a pluripotency state. Usp22 knockdown in ESCs lead to a decrease in differentiation and a transcriptional profile similar to ESCs. On the contrary, Usp22 overexpression leads to increased differentiation of ESCs into EBs even in the absence of stimuli that drives differentiation [52]. Another study in mice showed that Usp22 negatively regulates neuronal differentiation in the mouse developing brain. Usp22 was shown to deubiquitylate Hes1, a key transcriptional repressor for the maintenance of neuronal stem cell and progenitor cells, increasing its half-life [53].

## USP22 INVOLVEMENT IN PATHOLOGICAL CONDITIONS

Studies in human samples suggest that USP22 overexpression is involved in different pathological conditions such as cancer, neurological disorders, diabetes and male infertility. Glinsky et al published one of the first studies linking USP22 with poor cancer prognosis. They described that a 11-gene signature profile, which included USP22, was capable of predicting tumor recurrence, metastasis and poor survival after cancer diagnosis in several types of cancer: prostate, lung, breast, ovarian, bladder, lymphoma, glioma, mesothelioma, neuroblastoma, mantle cell lymphoma and acute myeloid leukemia. Some of the genes in this signature profile have been previously involved with processes such as mitosis and chromatin remodeling [54].

Several other studies have confirmed overexpression of USP22 at the protein level in different types of cancer as well as its correlation to poor patient survival [55, 56]. USP22 overexpression has been positively correlated with proteins involved in proliferation and negatively correlated with the expression tumor suppressor proteins. For example, USP22 positively correlated with the expression of BMI-1 and pAKT but negatively with PTEN in non-small cell lung carcinoma [57]. Ki67 expression was associated with USP22 overexpression in cervical cancer, prostate cancer and oral squamous cell carcinoma [40, 55, 58].

It is clear that USP22 is involved in tumor progression because it is not only increased at the mRNA level but also at the protein level. Moreover, USP22 expression correlates with proteins involved in cell growth and cell cycle. In fact, USP22 knockdown in different types of cancer cells, lead to cell cycle arrest in the G0/G1 phase and decreased in-vivo tumor growth [32, 59, 60]. Among the key players in driving tumor growth is c-MYC. USP22 was shown to be necessary for c-MYC transcriptional activity [59]. However, it is not known how USP22 affects c-MYC activity. It could be through a direct mechanism where USP22 deubiquitylates c-MYC inducing its stabilization/activation, or indirectly through ubiquitin removal from histones at c-MYC target genes, recruitment of other transcriptional machinery or deubiquitylation of proteins important for c-MYC activity.

In addition to the role of USP22 in the cell cycle, USP22 promotes the activity of the androgen receptor (AR). In prostate cancer cells, upon ligand binding, USP22 is recruited (and also promotes AR recruitment) to the promoter regions of AR- dependent genes such as KLK2, KLK3/PSA, TMPRSS2 and FKBP5. This increases the transcription levels of these genes for which USP22 deubiquitylase activity is necessary. In the presence of USP22, AR protein levels are increased in prostate cancer cell lines. Interestingly, this was not at the transcriptional level since AR mRNA levels did not change [40]. In addition, USP22 was shown to immunoprecipitate with AR indicating that AR is probably another USP22 substrate. In this regard, one study shows that USP22 has no effect on AR protein stability [24] but another one shows that USP22 indeed affects AR stability [40]. These differences might be due to different experimental conditions. Interestingly, AR ubiquitylation by RNF6 leads to changes in the expression of a subset of AR-target genes by modifying its recruitment to the promoter of these genes. RNF6 did not change AR stability but rather its activity and it is required for prostate cancer cell growth [61]. Therefore it is possible that RNF6 and USP22 contribute to prostate cancer progression by changing AR activity and stability.

A component of the SAGA DUB module, Ataxin 7 (Sgf73 in yeast), has been linked to an autosomal neurodegenerative disease called spinocerebellar ataxia 7 (SCA7). In this disease, Ataxin-7 protein contains additional poly-glutamine (polyQ) repeats (more than 35) at its N-terminal domain that leads to cerebellar and retinal degeneration [62]. Although it was debatable whether poly(Q) Ataxin-7 incorporated into the SAGA complex and decreased SAGA-meditated histone acetylation [63–66], other studies showed that poly(Q) Ataxin-7 increased H2B ubiquitylation with no changes in H3 acetylation [67, 68]. This indicated that poly(Q) Ataxin-7 likely decreased USP22 activity. Recently, Lan et al. reported that poly(Q) Ataxin-7 does not directly decrease USP22 enzymatic activity, but instead, it forms aggregates that impair USP22 binding to its substrates [68]. These results open the possibility that disruption of USP22 activity in SCA7 might be in part responsible for the phenotype observed in these patients.

## USP22 AS A POSSIBLE ONCOGENE

Based on the function and activity of USP22 mentioned above, it is possible that USP22 behaves as an oncogene. Although the frequency of USP22 somatic mutations reported is very low (<0.5%) [69], multiple reports show that USP22 is highly expressed at the mRNA and protein level in several types of cancer. USP22 controls cell growth and activation in different ways: it induces changes in promoter regions of genes by removing ubiquitin moieties from histones H2A and H2B leading to transcription activation; it induces cell cycle progression by the stabilization of cyclin B and TRF1 which induce cell mitosis and protection of telomeres during cell division; USP22 inhibits the activation of apoptotic pathways through the stabilization of Sirt1; and finally, USP22 induces activation of transcriptional factors such as c-MYC and AR. Moreover, it seems that USP22 may be involved in mRNA processing and splicing, although this has not yet been clearly established. Therefore, in cancer, USP22 up regulation leads to the abnormal activation of multiple pathways that are directed to cell survival. It is likely that USP22 overexpression in cancer is a downstream effect of the activation of pro-survival pathways. This makes USP22 an attractive therapeutic candidate since these multiple pathways will converge in the same protein.

## USP22 TARGETING IN CANCER THERAPY

USP22 is emerging as a potential oncogene linked not only to resistance and metastasis in different types of cancer. In addition, because USP22 is an enzyme, it is an attractive target for the development of molecules that can inhibit its catalytic activity. Due to the important roles that deubiquitylases have in cancer, efforts are being made to design small inhibitors for use as anti-cancer therapy. Some of these inhibitors target UCHL1, UCHL3, USP14, USP2 and USP7 [70].

One of the challenges of finding/designing inhibitors that target DUBs is their specificity since most DUBs are cysteine proteases and their inhibition can affect several other DUBs with similar active site [8]. However, new approaches are being explored such as the design of allosteric and exosite inhibitors, which could overcome this problem [71]. Interestingly, Chauhan et al [72], showed that a highly selective small inhibitor of USP7 (P5091) is capable of decreasing multiple myeloma growth in cell lines and xenograft models without affecting normal cells. In addition, P5091, besides working synergistically with conventional treatment drugs, induces cell death of multiple myeloma cells that are resistant to the standard therapy. Although the exact mechanism by which P5091 acts on inhibiting USP7 is not yet clear, this opens the door for targeting DUBs in cancer. Nonetheless USP22 inhibitors have not been identified yet, screening of small molecule inhibitors is currently ongoing [73].

## PERSPECTIVES AND FUTURE DIRECTIONS

USP22 has emerged as a new DUB linked to carcinogenesis and neurodegenerative disease. However, very little is known about its function and regulation in normal tissues or cancer. Most of the studies elucidating some of the targets and activity of USP22 have been performed in yeast. Few studies have been performed in mammalian cells and none have been performed using conditional knockout mouse models. Defining the role of USP22 in homeostasis and cancer will be necessary to establish whether targeting this DUB could be beneficial.
